# Robust Direction-of-Arrival Estimation Using Zero-Crossing-Based Time Delay Measurement for Navigation in GNSS-Denied Environments

**DOI:** 10.3390/s26051600

**Published:** 2026-03-04

**Authors:** Lin Lian, Shenpeng Li, Guojun Huang, Yang Wu, Qin Ren

**Affiliations:** Ordnance NCO Academy, Army Engineering University of PLA, Wuhan 430075, China; 13545342003@163.com (S.L.); hgj314687339@163.com (G.H.); 15871737692@139.com (Y.W.); renqin_1990@163.com (Q.R.)

**Keywords:** Loran-C, DOA estimation, TDOA, USBL array

## Abstract

This paper investigates Direction-of-Arrival (DOA) estimation of Long-Range Navigation-C (Loran-C) signals using an Ultra-Short Baseline (USBL) receiving array. Two least-squares angle estimation approaches based on inter-element delay measurements are examined, including Correlation-based Least-Squares (Corr-LS) and a Zero-Crossing-based Least Squares (ZC-LS). In both methods, relative delays are extracted only within the local array and subsequently mapped to azimuth through a geometric least squares formulation; the approach is, therefore, distinct from distributed time difference-of-arrival (TDOA) localization. For comparison, the Multiple Signal Classification (MUSIC) algorithm is implemented as a covariance-based DOA estimator that operates without explicit delay extraction. Experiments were conducted using Loran-C transmissions from the Xuancheng, Xi’an, and Rongcheng stations, with 100 valid pulse groups collected for each station. Statistical analysis using boxplots shows that Corr-LS exhibits the largest variance due to broadened or shifted correlation peaks, particularly under skywave–groundwave interference. ZC-LS reduces both variance and bias by exploiting the deterministic zero-crossing structure of the Loran-C waveform. MUSIC produces the most concentrated azimuth estimates but requires a well-conditioned covariance matrix and substantially higher computational costs. The results demonstrate that ZC-LS achieves a favorable balance among angular accuracy, robustness, and real-time feasibility, making it suited for compact Loran-C receivers and complementary navigation applications in GNSS-challenged environments.

## 1. Introduction

The Global Navigation Satellite System (GNSS) underpins modern navigation and timing infrastructure; however, its civil signals are inherently weak and, therefore, vulnerable to interference, jamming, and spoofing attacks. Ensuring resilient positioning, navigation, and timing (PNT) capability consequently requires complementary terrestrial systems that can operate independently of GNSS satellite line of sight. In addition to terrestrial low-frequency navigation systems, recent research has explored localization in GNSS-denied environments using signals of opportunity transmitted by Low Earth Orbit (LEO)satellites [1,2]. These approaches exploit measurements such as Angle of Arrival (AoA) [3] or Doppler shifts [4] for positioning without dedicated navigation payloads. While such techniques offer excellent global coverage and geometric diversity, they typically rely on satellite visibility and accurate orbital information. The Long-Range Navigation-C (Loran-C), employing high-power ground transmitters and robust low-frequency propagation characteristics, has been widely studied as a potential backup and complement to GNSS such as BeiDou and Global Positioning System (GPS) [5].

Loran-C navigation relies primarily on groundwave propagation, which provides stable timing and positioning performance over short to medium distances—typically several hundred kilometers under favorable surface conductivity. In addition, Loran-C signals may propagate as skywaves via ionospheric reflection, extending reception ranges beyond 1000 km. This extended coverage, however, introduces complex propagation delays and multipath distortions because ionospheric reflection height and refractive properties vary with local time, season, and solar–geomagnetic activity [6,7].

From a positioning perspective, sky-wave signals are difficult to exploit using conventional time-based multilateration techniques such as Time of Arrival (TOA) or time difference of arrival (TDOA). The highly variable ionospheric delay often results in large ranging errors unless sophisticated correction or estimation algorithms are applied. Considerable research has, therefore, focused on delay mitigation methods, including WRELAX-based models, sparse reconstruction, and hybrid signal processing strategies [8,9]. Despite these advances, reliable long-range positioning using timing information alone remains challenging in practical deployments.

An alternative strategy is to exploit spatial information provided by receiving arrays capable of direction-finding. Direction-of-Arrival (DOA) measurements are inherently less sensitive to absolute propagation delay and can remain informative, even when sky-wave components dominate or when multi-station synchronization is required for multilateration is unavailable. By combining angle measurements with geometric intersection or auxiliary timing information, array-based systems can improve localization robustness and operational flexibility, enhancing the viability of Loran-C as a complementary GNSS backup solution.

Motivated by this context, this work focuses on azimuth DOA estimation using an Ultra-Short Baseline (USBL) array for Loran-C signal reception. Unlike distributed TDOA localization approaches, the methods investigated here utilize inter-element delay extraction solely as an intermediate step for angle estimation within a compact receiver platform. The study evaluates correlation-based and zero-crossing-based delay estimators for a least squares direction-finding framework and compares them with covariance-based subspace estimation using Multiple Signal Classification (MUSIC). AoA estimation can be achieved using beamforming and phase interferometry techniques, as well as dedicated digital architectures for real-time implementation. Subspace-based methods such as MUSIC provide super-resolution capability by exploiting the covariance matrix eigenstructure. In this work, MUSIC is adopted because the extremely long wavelength of Loran-C signals necessitates an ultra-compact array, for which interferometric approaches suffer from degraded accuracy while MUSIC maintains superior resolution and robustness. Experimental measurements using operational transmitters clarify performance trade-offs among accuracy, robustness, and computational efficiency for practical GNSS-resilient navigation applications.

The primary contributions of this work are summarized as follows: (1) A zero-crossing-based delay extraction method designed for low-complexity real-time implementation. (2) A unified DOA estimation framework integrating orthogonal magnetic antenna data. (3) Comprehensive statistical evaluation of angular accuracy under experimental conditions. (4) Demonstration of applicability to navigation scenarios where GNSS signals are unavailable or unreliable.

## 2. Materials and Methods

In this study, a USBL array is employed to receive Loran-C groundwave signals for DOA estimation and intersection-based positioning. The array consists of *N* elements arranged in a cross configuration, with inter-element spacing d ≪ λ to ensure minimal spatial aliasing. Each element receives the same Loran-C pulse sequence, and the relative phase and time delay differences between channels are used to estimate the angle of arrival and position.

Let s(t) denote the transmitted Loran-C pulse and $x_{i}(t)$ represent the received signal at the *i*-th antenna element, where the index i denotes the antenna element number and satisfies $i\in \left\{1,\;2,\;\ldots ,\;N\right\}$ (*N* is the total number of antenna elements in the receiving array):

(1) $$x_{i}(t)=A_{i}\cdot s(t-\tau_{i})+n_{i}(t)$$

where $A_{i}\;$ is the channel attenuation coefficient of the *i*-th element, $\tau_{i}\;$ is the propagation delay relative to the reference element, and $n_{i}\left(t\right)\;$ is additive noise. The DOA *θ* is estimated by exploiting the time delay vector ${\tau =\left[\tau_{1},\tau_{2},\ldots ,\tau_{N}\right]}^{T}$.

### 2.1. MUSIC-Based DOA Estimation

The MUSIC algorithm is applied to achieve high-resolution DOA estimation [10]. The received data matrix is expressed as

(2) $$X={\left[x_{1}\left(t\right),x_{2}\left(t\right),\ldots ,x_{N}\left(t\right)\right]}^{T}$$

where the superscript T denotes the transpose operator. The covariance matrix of the received data is computed as:

(3) $$R_{x}=E\left\{{XX}^{H}\right\}$$

where $E\left\{\cdot \right\}$ represents the ensemble average and H denotes the Hermitian (conjugate transpose) operator. Through eigendecomposition, the signal subspace and noise subspace are separated:

(4) $$R_{x}=E_{s}\Lambda_{s}E_{s}^{H}+E_{n}\Lambda_{n}E_{n}^{H}$$

where $E_{s}$ is the signal subspace matrix, $\Lambda_{s}$ is the diagonal matrix of signal eigenvalues, $E_{n}$ denotes the noise subspace matrix, and $\Lambda_{n}\;$ is the diagonal matrix of noise eigenvalues.

The MUSIC spatial spectrum is then given by

(5) $$P_{MUSIC}\left(\theta \right)=\frac{1}{a^{H}(\theta )E_{n}E_{n}^{H}a(\theta )}$$

where a(θ) is the array steering vector. The DOA is estimated by finding the angle *θ* that maximizes $P_{MUSIC}\left(\theta \right)$ [10].

### 2.2. Least Squares Intersection Localization

Another method is least squares intersection localization based on a nonlinear array [11]. Unlike conventional linear arrays, the sensor elements in a nonlinear array are distributed in a two-dimensional or irregular geometry, which enables flexible installation and improved spatial coverage while maintaining a compact aperture.

Let the array consist of *N* receiving elements with known coordinates $r_{i}={\left[x_{i},y_{i},z_{i}\right]}^{T}\;$in the local reference frame. Assuming a plane wave incident from direction $k={\left[\;cos\;\theta \;cos\;\phi ,\;\;cos\;\theta \;sin\;\phi ,\;\;sin\;\theta \right]}^{T}$, where *θ* and *ϕ* denote the elevation and azimuth angles, respectively, the time delay at the *i*-th element relative to a reference sensor can be expressed as

(6) $$\tau_{i}=-\frac{1}{c}r_{i}^{T}k$$

where $c=3\times 10^{8}\;m/s\;$ (speed of light, approximated for groundwave propagation) and $r_{i}^{T}$ is the transpose of $r_{i}$. The measurable TDOA between any two elements *i* and *j* is then

(7) $$\Delta \tau_{ij}=\frac{1}{c}\;{\left(r_{j}-r_{i}\right)}^{T}k$$

Given a set of measured TDOA as $\Delta \tau_{ij}$, the direction vector ***k*** can be estimated by solving the following least squares problem:

(8) $$\min{\left[\Delta \tau_{ij}-\frac{1}{c}{\left(r_{j}-r_{i}\right)}^{T}k\right]}^{2}$$

subject to ||***k***|| = 1.

Once the direction vector is obtained, the azimuth angle *θ* and elevation angle *ϕ* are derived as

(9) $$\theta ={tan}^{-1}\;(\frac{k_{y}}{k_{x}}),\phi ={tan}^{-1}\;(\frac{k_{z}}{\sqrt{k_{x}^{2}+k_{y}^{2}}})\;$$

where $k_{x}$, $k_{y}$, and $k_{z}$ are the x,y,z components of the incident direction vector k, corresponding to the projection of the plane wave propagation direction onto the local coordinate axes.

This approach allows accurate DOA estimation, even for irregularly spaced sensors, avoiding the geometric constraints of linear arrays. Furthermore, when combined with correlation-based or zero-crossing-based delay estimation, the nonlinear USBL configuration provides a robust and compact solution for real-time Loran-C angle estimation and localization. When only the groundwave component is considered, the elevation angle *ϕ* can be assumed to be zero.

Loran-C signals, operating at low frequencies (~100 kHz) with long wavelengths (~3 km), can be approximated as plane waves over practical reception distances. Given that the propagation is predominantly along the Earth’s surface, the wave incidence at the receiver can be assumed to have a negligible elevation angle (i.e., near 0°), allowing the plane wave assumption to simplify modeling of amplitude, phase, and AoA.

### 2.3. Time Delay Estimation

For validation and comparison, time delay differences between array elements are also extracted using two classical methods.

#### 2.3.1. Cross-Correlation Method

The time delay Δτ between two signals $x_{1}\left(t\right)\;$and $x_{2}(t)$ can be estimated by maximizing the normalized cross-correlation function:

(10) $$R_{12}\left(\tau \right)=\frac{\int_{-\infty }^{+\infty }x_{1}\left(t\right)x_{2}\left(t-\tau \right)dt}{\sqrt{\int_{-\infty }^{+\infty }x_{1}^{2}\left(t\right)dt\cdot \int_{-\infty }^{+\infty }x_{2}^{2}\left(t\right)dt}}$$

A window length of 10 ms (covering one complete Loran-C pulse cycle) is used for the correlation calculation. The cross-correlation function in MATLAB R2025a (The MathWorks, Inc., Natick, MA, USA) can be used to estimate the normalized cross-correlation $R_{12}\left(\tau \right)$. The delay estimate is given by:

(11) $$\hat{\tau }=\arg\max R_{12}\left(\tau \right)$$

This approach is robust under high Signal-to-Noise Ratio (SNR) conditions but is sensitive to multipath distortions.

#### 2.3.2. Zero-Crossing Detection Method

Given the highly repetitive nature of Loran-C pulses, the zero-crossing time of the envelope or carrier is used to determine the time difference between signals. After filtering and normalization, the third positive-to-negative zero-crossing of the first pulse in each Loran-C pulse group is designated as the reference (occurring after the initial transient response and before strong skywave contamination) [12]. The time offset (TDOA) between two elements *i* and *j* is computed as:

(12) $$\Delta \tau =t_{z,j}-t_{z,i}$$

where $t_{z,i}\;$ and $t_{z,j}$ are the reference zero-crossing times of the *i*-th and *j*-th elements, respectively. This method is simple and suitable for real-time applications. In our work, zero-crossing detection is used as an intermediate measurement for DOA estimation, rather than for hyperbolic positioning.

## 3. Experimental Setup

### 3.1. Receiving Array and Hardware Configuration

The receiving system was deployed in Wuhan, China. The array consists of four spatially separated sensor elements arranged in a cross geometry, forming a USBL configuration suitable for low-frequency direction finding. Each sensor element is an orthogonal square-loop coil with 100 turns and a side length of 25 cm, capable of capturing the horizontal magnetic field components of the incoming Loran-C signals. A sampling frequency of 100 MHz was adopted. The four antennas were connected to the receiver using identical-length cables, and all receiver channels were driven by a common clock. Consequently, inter-channel timing synchronization errors are assumed to be negligible.

**Figure 1:**
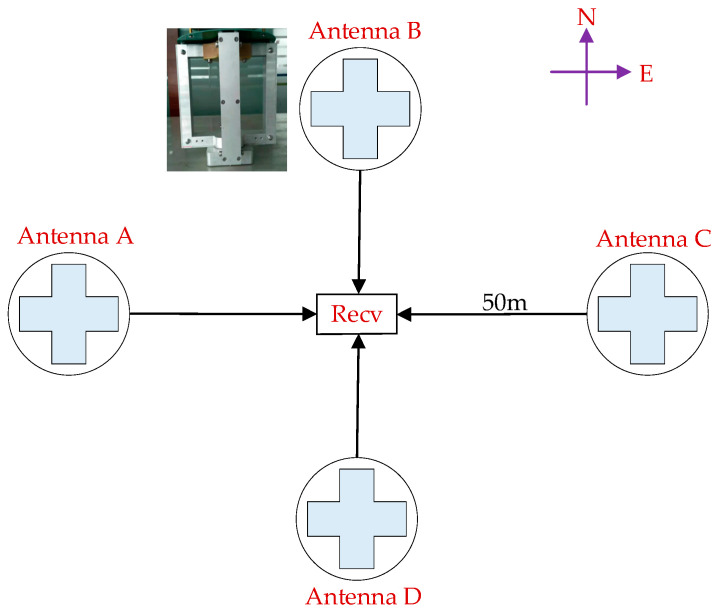
Schematic diagram of the four-element array geometry relative to the receiver reference point. The black lines represent the signal cables, and the direction of the arrows indicates the signal transmission direction from each antenna to the receiver (Recv).

The four elements are symmetrically placed around the receiver reference point, each at a radial distance of 50 m. They are labeled as A, B, C, and D according to their spatial positions. A is located to the west of the receiver center, B to the north, C to the east, and D to the south, as illustrated in Figure 1.

Loran-C signals feature a wavelength on the order of several kilometers, making a λ/2 element spacing infeasible for real-world deployment due to an excessively large physical aperture—especially for our target shipborne platforms that demand compact antenna layouts. We thus employ a USBL configuration with element spacing far smaller than the signal wavelength. While this deviates from the conventional λ/2 design principle for side-lobe level control, it enables practical implementation while still supporting AoA estimation via inter-channel phase differences. This small spacing confines inter-element phase differences within a limited range, thereby effectively eliminating spatial aliasing and phase ambiguity in AoA estimation.

All four sensors are connected to a multi-channel receiver through cables of equal electrical length to ensure phase consistency across channels. This geometric configuration provides a well-defined baseline structure for phase difference estimation and improves the numerical conditioning of DOA solutions in subsequent processing.

Since the array aperture is very small, potential mutual coupling between closely spaced elements may affect the array response. Orthogonal loop antennas and careful placement were employed to mitigate mutual coupling [13]; however, residual coupling effects are not explicitly modeled in this work and will be addressed in future studies.

### 3.2. Signal Sources and Observation Geometry

Three Loran-C transmitting chains were selected as signal sources. The stations belong to the Xuancheng (GRI 8390), Xi’an (GRI 6000), and Rongcheng (GRI 7430) chains, located at (30.9° N, 118.7° E), (34.26° N, 108.94° E), and (37.15° N, 122.45° E), respectively. The Group Repetition Interval (GRI) is the time interval between two consecutive pulse groups, acting as a unique identifier for different Loran-C transmitting chains (independent of carrier frequency).

**Figure 2:**
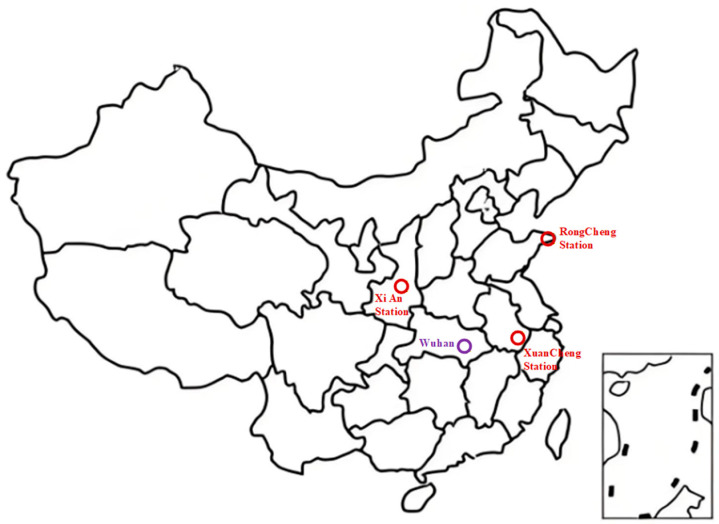
Spatial distribution of the Wuhan receiving station and the Xuancheng, Xi’an, and Rongcheng Long-Range Navigation-C (Loran-C) transmitting stations. The red circles indicate the positions of the Loran-C transmitting stations, and the purple circle denotes the position of the receiver located in Wuhan.

The receiving array was deployed in Wuhan. The true azimuths from the receiving site to the Xuancheng, Xi’an, and Rongcheng stations are 84.23°, 310.61°, and 43.23°, respectively, which are used as reference values for evaluating direction-finding accuracy. The spatial distribution of the receiving site and the three transmitting stations is illustrated in Figure 2.

### 3.3. Phase Extraction Approaches

Two phase extraction strategies were applied to the received multi-channel signals. The first method implements zero-crossing detection to estimate relative phase differences between sensors along the Loran-C pulse envelope. The second method utilizes cross-correlation to estimate inter-channel phase delays with improved noise robustness. Both sets of extracted phase differences are subsequently used as inputs to the DOA estimation algorithms.

### 3.4. DOA Estimation Procedures

The initial DOA is estimated using the MUSIC algorithm applied to the measured covariance matrix of the array data. The MUSIC spatial spectrum is searched over the azimuth domain to locate the main arrival direction. To refine the estimate, a least squares procedure is then applied to the extracted phase differences and the array geometry model, yielding the final azimuth estimate. The use of both MUSIC and least squares processing enables the combination of high-resolution subspace estimation and direct physical-model-based angle refinement.

## 4. Results and Discussion

It should be noted that this study evaluates angular estimation performance rather than full position estimation. The presented statistical results characterize DOA accuracy, which forms the basis for localization through bearing intersection or hybrid fusion with timing measurements.

### 4.1. DOA Estimation Based on Cross-Correlation

**Figure 3:**
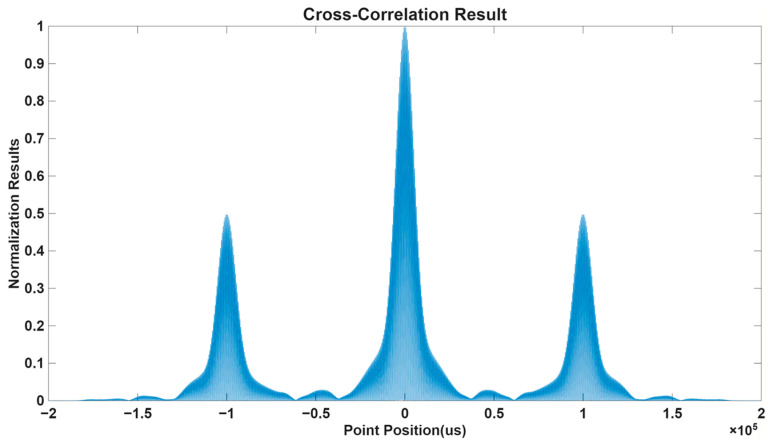
Correlation results obtained from Loran-C signal reception using the cross-correlation method.

Time difference measurements were first estimated using the cross-correlation method. The correlation output for the Loran-C signals received is shown in Figure 3. The correlation waveform exhibits a clear periodic structure, which corresponds to the carrier-frequency component embedded in the Loran-C signal. In addition, impulsive characteristics can be observed due to the pulse-modulated nature of Loran-C transmissions.

However, the correlation peaks are relatively broad. This broadening effect reduces the temporal resolution of the correlation estimator and introduces uncertainty in locating the true delay peak. As a result, the extracted inter-element time differences contain noticeable biases, which propagate into the DOA estimation stage.

The extracted time differences were fed into the least squares DOA estimation model to compute the azimuth angles corresponding to the three transmitting stations. The estimated azimuth angles and their deviations from the true values are summarized in Table 1. Antennas A, B, C, and D denote the west, north, east, and south elements of the receiving array, respectively.

The results indicate that the correlation-based time difference extraction leads to azimuth estimation errors typically between 3° and 11°. The primary cause of this error is the broadened correlation peak, which limits delay resolution. Moreover, the peak broadening increases susceptibility to noise and makes the estimator highly sensitive to waveform distortion caused by ionospheric propagation [14,15].

This demonstrates that the correlation method is insufficient for high-precision DOA estimation in very-low-frequency Loran-C reception, especially when using USBL arrays where time delays are small and delay errors directly amplify angular errors.

### 4.2. Zero-Crossing Method for DOA Extraction

**Figure 4:**
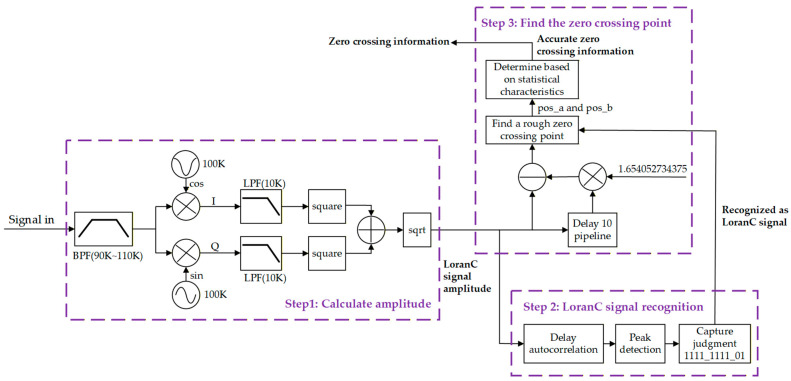
Procedure of the zero-crossing technique applied to the first pulse of each Loran-C pulse group for improved time difference-of-arrival (TDOA) measurement. Arrows indicate the direction of data flow.

**Figure 5:**
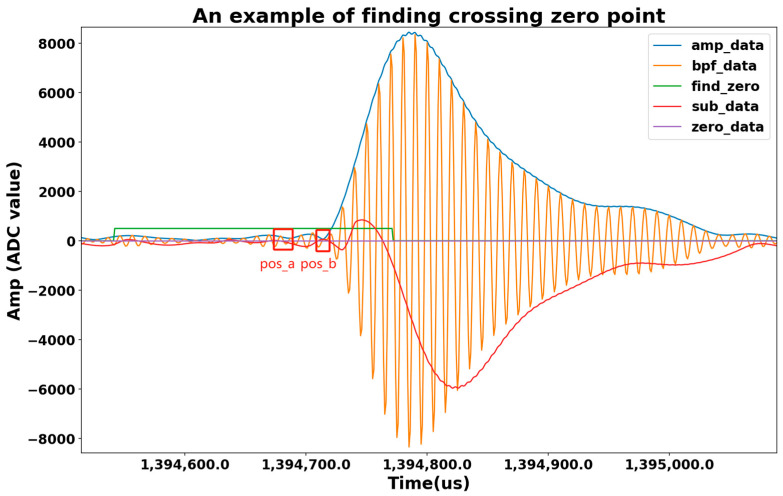
Illustration of amplitude-based zero-crossing determination for Loran-C signals, showing selection between two candidate positions.

In addition, a zero-crossing method was employed to improve TDOA extraction and direction finding. The third zero-crossing of the first pulse of each Loran-C pulse group was designated as the reference zero-crossing, as this zero-crossing occurs after the initial transient response and before strong skywave contamination, making it less sensitive to multipath distortion and noise. The procedure is illustrated in Figure 4. The received signals were first band-pass filtered and amplitude-demodulated via coherent detection. Noise filtering was performed using a 512th-order Finite Impulse Response (FIR) filter. The amplitude signals were then autocorrelated, and signals from different GRIs were identified. Two candidate positions for the third zero-crossing were initially determined based on amplitude peaks, and statistical analysis of the intervals preceding the third zero-crossing (typically 22.5 μs and 32.5 μs) was used to select the correct zero-crossing position. Figure 5 shows an example, where Pos_a and Pos_b are candidate positions and Pos_b is confirmed as the correct zero-crossing.

### 4.3. Analysis of Cross-Correlation and Zero-Crossing Method

The TDOA-based direction-finding results using the correlation method and the zero-crossing method are summarized in Table 1 and Table 2. Both methods utilize the four-element cross-shaped array (A, B, C, D) to estimate the azimuth angles of Loran-C signals from three stations (Xi’an, Rongcheng, and Xuancheng).

Comparison between the two methods reveals several key points.

1. Accuracy:

The zero-crossing method consistently achieves smaller mean azimuth errors compared to the correlation method. For example, Xi’an station shows an average reduction of approximately 2–3° in absolute error.

2. Stability:

The zero-crossing method produces less fluctuation across multiple measurements. In contrast, the correlation method results display larger variability, especially for stations farther away from the receiver.

3. Noise Robustness:

The zero-crossing method is less affected by amplitude modulation artifacts and background noise because it relies on the timing of characteristic signal zero-crossings rather than the broad autocorrelation peaks.

4. Station Dependence:

Both methods show slightly larger errors for Xuancheng station, likely due to longer propagation distances and associated multipath effects. Rongcheng station, at intermediate distance, shows relatively small errors for both methods, indicating a dependence of accuracy on propagation path characteristics.

In conclusion, while both methods are capable of providing azimuth estimates for Loran-C signals using a USBL array, the zero-crossing method demonstrates superior performance in terms of accuracy, stability, and noise resilience. This makes it the preferred approach for practical TDOA-based direction-finding applications, especially under conditions of weak signals or significant skywave interference.

## 5. Comparison of ZC-LS, Corr-LS, and MUSIC DOA Estimation Using Xi’an Station Data

**Figure 6:**
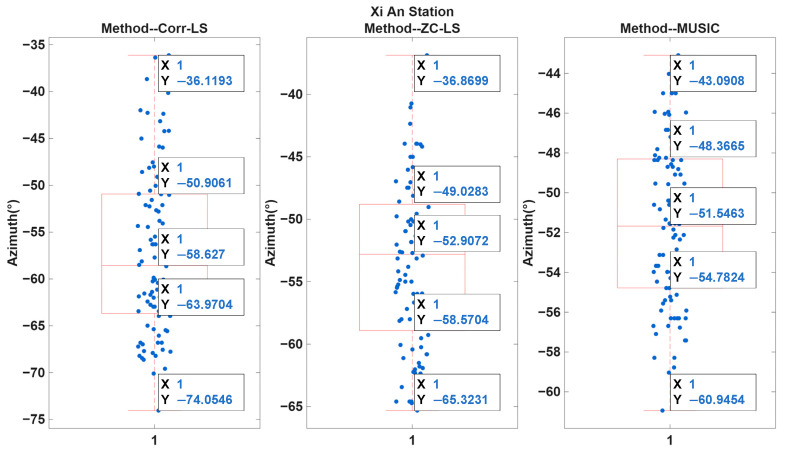
Boxplot comparison of Correlation-based Least-Squares (Corr-LS), Zero-Crossing-based Least-Squares (ZC-LS), and Multiple Signal Classification (MUSIC) methods for 100 Loran-C pulse group measurements at Xi’an station. The blue dots represent the raw azimuth measurements from each trial, while the red boxes show the statistical distribution of the data, including the median, 25th and 75th percentiles, and whiskers.

To further evaluate the performance of the proposed ZC-LS method, a comparative analysis was carried out among the Corr-LS, ZC-LS, and MUSIC algorithms. A total of 100 valid Loran-C pulse group measurements were collected at the Xuancheng, Xi’an, and Rongcheng stations. The dataset was filtered to ensure that clear and accurate zero-crossings were present prior to processing. Figure 6 shows the boxplot comparison of the three methods for Xi’an station.

For Xi’an station, the six characteristic statistical values (limit inferior, lower quartile, median, upper quartile, limit superior, and Root Mean Square Error (RMSE) are summarized in Table 3.

From the boxplot, the Corr-LS method exhibits the largest spread, with a large interquartile range and notably extremely lower whisker values. This indicates that Corr-LS suffers and is highly sensitive to pulse envelope distortion and skywave interference, which is consistent with the fact that this method relies on delay estimation extracted from cross-correlation peaks. When skywave echoes overlap with the groundwaves, the correlation peaks become broadened or shifted, leading to greater estimation bias and variability.

The ZC-LS results show a significant reduction in both median deviation and variance compared to Corr-LS. The interquartile range becomes noticeably narrower, and the lower whisker increases from approximately −74° to −65°, indicating robustness. This improvement is attributed to the fact that the zero-crossing structure of the Loran-C waveform is more deterministic and less affected by amplitude fluctuations, enabling the least squares fitting to effectively suppress multipath-induced bias in the time delay estimates. However, a small number of outliers still exist, reflecting zero-crossing detection that remains sensitive to noise at specific portions of the pulse envelope.

The MUSIC algorithm shows the most concentrated distribution, with the smallest variability among the three methods. The median value (≈−51.55°) is close to the expected azimuth direction, and both quartiles are tightly clustered around the median. This improvement is attributed to MUSIC’s subspace-based decorrelation capability, which separates the dominant signal components from residual noise. However, MUSIC does not outperform ZC-LS uniformly in all scenarios. Since Loran-C has limited effective bandwidth and non-ideal coherence, its covariance matrix is sometimes low-rank or poorly conditioned, limiting MUSIC’s theoretical resolution advantage. Furthermore, MUSIC requires a higher computational load, making it less suitable for embedded or real-time implementations.

Table 4 shows the boxplot statistics of the DOA estimation results at the remaining two stations using the three methods. Based on the boxplot results obtained from the Xi’an, Xuancheng, and Rongcheng stations, the performance of the three DOA estimation methods can be further compared under different incoming signal directions. Although all three stations exhibit similar rankings in terms of estimation stability (MUSIC being the most concentrated, followed by the ZC-LS method, and then the Corr-LS method), the degree of performance variation varies across the three stations, indicating that the propagation geometry and signal composition influence the effectiveness of each method.

At Xi’an station, the median azimuth estimates are approximately −58.6° for Corr-LS, −52.9° for the zero-crossing method, and −51.5° for MUSIC. The Corr-LS method displays the largest spread, with the lower whisker reaching −74°, indicating severe instability under complex wavefield conditions. This is attributed to the reliance on correlation peak detection, which is highly susceptible to skywave–groundwave overlap and pulse envelope distortion. The zero-crossing method substantially narrows the interquartile range, demonstrating improved robustness since the zero-crossing structure is more deterministic and less affected by amplitude variations. MUSIC shows the smallest dispersion, suggesting successful subspace separation. However, it should be noted that this advantage depends on stable signal covariance estimation, which may not always be guaranteed for Loran-C signals with limited effective bandwidth.

In contrast, the Xuancheng station results show that all three methods produce direction estimates closer to the theoretical incoming azimuth (~80°). MUSIC again exhibits the tightest clustering, indicating that the signal at this station has dominant groundwave components and higher waveform coherence, which benefits subspace-based estimation. Although the correlation method still displays a comparatively higher dispersion, the absence of extreme outliers suggests that improved propagation stability mitigates correlation peak ambiguity. This highlights that the performance of Corr-LS is strongly dependent on the stability of the propagation channel.

For Rongcheng station, the differences among the three methods are further diminished. The median values lie closely between 38° and 42°, and the upper quartiles of the zero-crossing method and MUSIC nearly coincide. This indicates that the waveform structure at this station remains consistent across measurements, and skywave interference is relatively weak. Under such conditions, the zero-crossing method yields performance approaching that of MUSIC. The correlation method, however, continues to show the largest variational range, reaffirming its sensitivity to even moderate levels of waveform distortions.

Loran-C signals present specific characteristics that affect the performance of subspace-based DOA estimation algorithms, such as MUSIC.

MUSIC relies on an accurate estimation of the covariance matrix of the received signals, which is obtained by averaging over multiple temporal snapshots. For Loran-C, the low-frequency nature and long symbol duration limit the number of independent snapshots that can be collected within a given observation period. Insufficient snapshots lead to a poorly estimated covariance matrix, reducing the resolution and accuracy of the MUSIC algorithm. Therefore, careful selection of the snapshot number is critical to balance estimation accuracy and computational cost. Loran-C signals transmitted from different stations may exhibit partial coherence due to simultaneous propagation and multipath reflections, especially over conductive ground and sea surfaces. Coherent or highly correlated sources violate the standard MUSIC assumption of uncorrelated sources, causing the covariance matrix to be rank-deficient and leading to performance degradation. Spatial smoothing or forward–backward averaging techniques are often required to decorrelate coherent Loran-C signals prior to MUSIC application. Loran-C has a very narrow bandwidth (typically ~10 kHz), resulting in a long coherence time but limited frequency diversity. Narrowband signals simplify the use of standard MUSIC, which assumes narrowband sources. However, narrow bandwidth also limits temporal resolution, making it difficult to resolve closely spaced multipath arrivals or rapidly varying channels. The narrow bandwidth also affects the effective array aperture in frequency domain extensions of MUSIC, potentially limiting angular resolution.

## 6. Conclusions

This work presents a comparative study of TDOA-based least squares DOA estimation and direct MUSIC-based DOA estimation for Loran-C signals. The Corr-LS method is shown to be highly sensitive to skywave interference and pulse envelope distortions, resulting in significant estimation dispersion. The proposed ZC-LS method significantly improves estimation stability by utilizing the intrinsic zero-crossing structure of Loran-C pulses to obtain precise inter-element timing, thereby reducing both bias and variance across all three stations tested. The MUSIC algorithm provides the most concentrated estimation distribution.

In addition to estimation accuracy, computational efficiency is an important consideration for practical deployment in compact or real-time receivers. The three evaluated methods exhibit distinct complexity characteristics. The Corr-LS approach requires computation of cross-correlations between sensor pairs, with complexity proportional to the signal length and number of sensors, typically on the order of O(NL). This step dominates processing costs, particularly when long observation windows are used to stabilize peak detection. The proposed ZC-LS method replaces correlation with zero-crossing feature extraction and linear least squares solving. Zero-crossing detection scales linearly with signal length, and the least squares solution involves only small matrix operations. Therefore, its overall complexity remains approximately O(N), making it suitable for low-power implementation. In contrast, the MUSIC algorithm involves covariance matrix construction and eigenvalue decomposition. The eigendecomposition step scales cubically with array size, typically (O(M^3^)), and requires sufficient snapshots for stable estimation. This results in a substantially higher computational burden compared to the TDOA-based approaches. Overall, Corr-LS and ZC-LS are computationally lightweight, while MUSIC provides higher angular resolution at the expense of significantly increased processing cost.

Overall, the ZC-LS method achieves the best balance among accuracy, robustness, and real-time implementation requirements, making it particularly suitable for embedded and portable Loran-C direction-finding receivers. These results support the feasibility of using compact Loran-C arrays as a resilient terrestrial complement to GNSS, enhancing system performance under interference, jamming, or signal outages.

## Figures and Tables

**Table 1:** Azimuth estimation results using correlation-based time differences.

Station	Num	A	B	C	D	Azimuth	Azimuth Error
Xi’an Station	1	0	4	30	27	−52.5238	−3.1338
	2	0	6	31	27	−55.8855	−6.4955
	3	0	4	34	27	−55.9228	−6.5328
	4	0	3	30	27	−51.3402	−1.9502
	5	0	6	39	30	−58.3925	−9.0025
Rongcheng Station	1	34	0	2	25	52.0013	8.7713
	2	35	0	3	35	42.4362	−0.7938
	3	30	0	4	35	36.6071	−6.6229
	4	30	0	2	34	39.4725	−3.7575
	5	39	0	4	26	53.3929	10.1629
Xuancheng Station	1	35	19	0	27	77.125	−7.105
	2	38	19	0	19	90	5.77
	3	43	17	0	30	73.1786	−11.0514
	4	41	19	0	31	73.6861	−10.5439
	5	43	16	0	27	75.6507	−8.5793

**Table 2:** Azimuth estimation results using zero-crossing time differences.

Station	Num	A	B	C	D	Azimuth	Azimuth Error
Xi’an Station	1	0	3	38	29	−55.6197	−6.2297
	2	0	4	32	27	−54.2933	−4.9033
	3	0	4	28	29	−48.2397	1.1503
	4	0	4	30	30	−49.0856	0.3044
	5	0	5	31	26	−55.8855	−6.4955
Rongcheng Station	1	31	0	2	26	48.1221	4.8921
	2	31	0	3	26	47.1211	3.8911
	3	33	0	3	32	43.1524	−0.0776
	4	33	0	3	25	50.1944	6.9644
	5	30	0	2	28	45	1.77
Xuancheng Station	1	39	18	0	20	87.0643	2.8343
	2	36	18	0	21	85.2364	1.0064
	3	41	17	0	24	80.3112	−3.9188
	4	35	17	0	25	77.125	−7.105
	5	40	17	0	19	87.1376	2.9076

**Table 3:** Boxplot statistical metrics of the three Direction-of-Arrival (DOA) estimation methods at Xi’an station differences.

Station	Method	Limit Inferior	Lower Quartile	Median	Upper Quartile	Limit Superior	Root Mean Square Error (RMSE)
Xi’an Station	Corr-LS	−74.0546°	−63.9704°	−58.627°	−50.9645°	−36.1193°	11.8941
	ZC-LS	−65.3231°	−58.5704°	−52.9072°	−49.0283°	−36.8699°	7.8323
	MUSIC	−60.9454°	−54.7824°	−51.5463°	−48.3665°	−43.0908°	4.7003

**Table 4:** Statistical boxplot parameters of the three DOA estimation methods at Xuancheng and Rongcheng stations.

Station	Method	Limit Inferior	Lower Quartile	Median	Upper Quartile	Limit Superior	RMSE
Xuancheng Station	Corr-LS	53.6732°	68.5523°	74.7449°	82.9987°	98.1301°	12.9887
	ZC-LS	64.0557°	74.8459°	80.7274°	86.0995°	94.7636°	8.0571
	MUSIC	74.0546°	80.2724°	83.211°	85.9144°	91.5074°	4.1378
Rongcheng Station	Corr-LS	18.8861°	30.9638°	37.7468°	43.1524°	59.0362°	10.2018
	ZC-LS	23.3852°	34.992°	41.3086°	45°	55.2222°	7.1997
	MUSIC	30.4655°	38.4181°	42.1844°	45°	50.1944°	4.4127

## Data Availability

Dataset available on request from the authors. The raw data supporting the conclusions of this article will be made available by the authors on request.

## Abbreviations

The following abbreviations are used in this manuscript:

DOA	Direction of Arrival
Loran-C	Long-Range Navigation-C
USBL	Ultra-Short Baseline
Corr-LS	Correlation-based Least Squares
ZC-LS	Zero-Crossing-based Least Squares
TDOA	Time Difference of Arrival
MUSIC	Multiple Signal Classification
GNSS	Global Navigation Satellite System
PNT	Positioning, Navigation, and Timing
LEO	Low Earth Orbit
GPS	Global Positioning System
TOA	Time of Arrival
AoA	Angle of Arrival
GRI	Group Repetition Interval
SNR	Signal-to-Noise Ratio
RMSE	Root Mean Square Error
